# Activation of aortic baroreceptors depresses the somato–lumbar sympathetic reflex, reducing hindlimb muscle contractile force

**DOI:** 10.1016/j.jphyss.2025.100051

**Published:** 2025-11-20

**Authors:** Nobuhiro Watanabe, Kotaro Takeno, Naoko H. Tomioka, Masamichi Moriya, Hiroshi Nishimune, Harumi Hotta

**Affiliations:** a Department of Autonomic Neuroscience, Tokyo Metropolitan Institute for Geriatrics and Gerontology, Itabashi-ku, Japan; b Laboratory of Neurobiology of Aging, Tokyo Metropolitan Institute for Geriatrics and Gerontology, Itabashi-ku, Japan; c Department of Applied Biological Science, Tokyo University of Agriculture and Technology, Fuchu-shi, Japan

**Keywords:** Skeletal muscle, Sympathetic nerve activity, Neuromuscular junction, Aortic depressor nerve, Baroreceptor reflex, Immunohistochemistry

## Abstract

We investigated whether the same sympathetic nerves innervate arteries and neuromuscular junctions and aortic baroreceptor stimulation reduces muscle force in rats. Histological analysis with fluorescent labeling revealed nicotinic acetylcholine receptors, motor nerves, adrenergic nerves, and arteries in gastrocnemius muscle tissue. Whole-mount preparations revealed a potential extension of adrenergic nerves around an artery connected to the neuromuscular junction. Physiological analysis under anesthesia was conducted to examine the effects of baroreceptor activation on muscle contractility and contraction-induced lumbar sympathetic reflex discharges following tetanic motor nerve stimulation. Intravenous phenylephrine injection increased mean arterial pressure to ∼150 mmHg and reduced both tetanic force and sympathetic reflex discharges when the aortic depressor nerves were intact. Bilateral aortic nerve denervation nearly abolished these effects. These findings indicate that aortic baroreceptor afferent signaling decreases hindlimb muscle contractility, most likely by inhibiting the contraction-induced sympathetic reflex. Sympathetic nerves distributed to arteries and neuromuscular junctions may underlie this modulation.

## Highlights

• Baroreceptors and somatosensory inputs reflexively influence muscle sympathetic nerves.
• Sympathetic nerves at the neuromuscular junction (NMJ) may support motor nerve function.
• Histological analysis revealed sympathetic fibers on an artery connected to the NMJ.
• Aortic baroreceptor excitation reduces muscle contractility and sympathetic reflexes.
• Interaction between somatic and aortic afferents significantly modulates muscle force.

## Graphical Abstract

**Figure:**
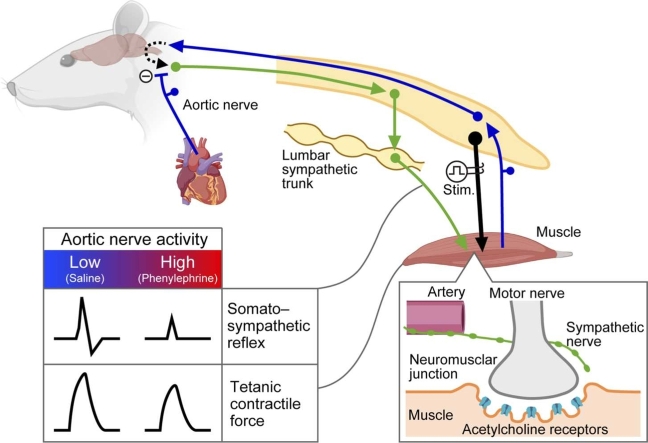

## Introduction

The sympathetic nervous system has spontaneous (or tonic) discharge activity [1], which is reflexively inhibited by baroreceptor afferent excitation [2], [3], [4], [5]. Conversely, motor nerve stimulation to contract limb muscles elicits sympathoexcitatory effects via the supraspinal pathway by group III and IV muscle afferents [6], [7], [8]. Functional co-activation of arterial baroreceptor– and somato–sympathetic reflexes may occur under conditions involving somatic afferent stimulation of muscle mechanoreceptors and metabolic receptors that respond to tetanic muscle contraction, such as during exercise and postural changes.

In hindlimb muscular nerves, such as the gastrocnemius and soleus nerve, approximately 90 % of all sympathetic fibers identified by stimulation of the lumbar sympathetic trunk (LST) show spontaneous discharge at rest that are reflexively modulated by somatosensory and aortic depressor nerve stimulation [9]. Based on muscle blood flow measurements, these muscle sympathetic nerve fibers are considered as vasoconstrictor nerves [10], [11], [12]. However, recent studies suggest that muscle sympathetic nerves innervate the neuromuscular junction (NMJ) [13], [14], [15], [16], [17], [18]. For example, in immunohistochemistry, tyrosine hydroxylase (TH), a marker of adrenergic nerves [13], [14], [16], [17], [18], and β2-adrenergic receptors [13], [19] have been labeled in the NMJ. However, adrenergic nerves may just pass near to NMJs, and adrenergic receptors can bind to blood catecholamines. Nonetheless, our recent study [20] showed the existence of presynaptic and postsynaptic proteins of sympathetic nerves at NMJ, namely sympathetic nerves detected by anti-TH antibody colocalized with a presynaptic protein, i.e., vesicular monoamine transporter 2. These two signals surrounded motor nerve terminals and nicotinic acetylcholine receptor (nAChR) clusters, whereas β2-adrenergic receptors colocalized with motor nerve terminals and appeared at a lower density in the extrasynaptic sarcolemma, indicating that adrenergic sympathetic nerves make synaptic contacts with both motor nerve terminals and the sarcolemma.

Moreover, functional studies suggested that muscle sympathetic nerves help maintain muscle force and mass, motor nerve efficiency, and NMJ morphology [13], [14], [15], [16], [17], [18], [21], [22]. For example, the tetanic force of hindlimb muscles produced by motor nerve stimulation decreased by ∼10 % after acute LST transection. In addition, transection of lumbar dorsal roots or cervical spinal cord resulted in a decrease in tetanic force comparable to LST transection, and supraspinal excitatory reflex discharges were recorded in lumbar sympathetic postganglionic fibers to the hindlimb in response to tetanic muscle contraction. These results indicate that the sympathetic reflex discharge induced by muscle contraction (hereafter abbreviated as an excitatory musculo–lumbar sympathetic reflex) facilitates motor nerve function [21], [22].

Taken together, we hypothesized that the same sympathetic nerves that innervate arteries also innervate NMJs. Consequently, arterial baroreceptor activity affects muscle force. Thus, this study aimed to histologically and physiologically clarify whether muscle force is affected by baroreceptor activation, which is stimulated by increasing blood pressure pharmacologically in anesthetized rats. Our results suggested that the aortic nerve is involved in muscle weakness with increased blood pressure, presumably by suppressing the musculo–lumbar sympathetic reflex. Therefore, postganglionic sympathetic nerve activity to the hindlimb was recorded to examine the extent of inhibition of the musculo–lumbar sympathetic reflex elicited in response to contractions by arterial baroreceptor activation.

## Methods

### Ethical approval

All experiments were performed following the “Guidelines for Proper Implementation of Animal Experiments” established by the Japan Society for the Promotion of Science in 2006 and approved by the Animal Care and Use Committee of the Tokyo Metropolitan Institute for Geriatrics and Gerontology (approval number: 23028). The investigators have acknowledged and adhered to the ethical principles outlined by the journal.

### Animals

The experiments were performed on 16 adult (2–6-month-old) male Fischer 344 rats (body weight, 205–358 g). Of the 16 rats, 4 were used for histological analysis and 12 for *in vivo* physiological experiments. The animals were purchased from Japan Charles River (Yokohama, Japan) and kept at the Tokyo Metropolitan Institute for Geriatrics and Gerontology. They were housed in individually ventilated cages (up to three animals per cage), with ad libitum access to a commercial pelleted diet (CRF-1, Oriental Yeast Co., Ltd., Tokyo, Japan) and filtered tap water with 2 ppm chlorine. The vivarium was maintained at 22°C ± 1°C and 50 % ± 10 % relative humidity under a 12-h light–dark cycle (lights off at 20:00 h).

### Histological analysis

Whole-mount specimens were prepared to examine the connection of sympathetic nerve fibers between the artery and NMJ. To visualize arteries with α-elastin, Alexa Fluor™ 633 Hydrazide (2 mg/kg, A30634, Thermo Fisher Scientific, MA, USA) was administered intravenously under transient sevoflurane anesthesia. After 3.5 h [23], animals were euthanized by an isoflurane overdose and fixed transcardially using 2 % paraformaldehyde/phosphate-buffered saline (PBS) solution before excising the gastrocnemius muscle. Whole-mount immunohistochemical staining of the gastrocnemius muscle was performed as previously described [24]. Muscles were fixed in 2 % paraformaldehyde/PBS, washed in PBS, sliced into four pieces using surgical blades, permeabilized in 0.5 % Triton/0.1 M glycine/PBS overnight at 4°C, and blocked for 24 h at 4°C in 2 % bovine serum albumin/2 % normal goat serum/0.5 % Triton/PBS. Tissues were incubated with primary antibodies (anti-TH, anti-neurofilament, and anti-synaptic vesicle glycoprotein 2 A (SV2); Table 1) for 72 h at 4°C and washed with PBS. Thereafter, whole-mount tissues were incubated with secondary antibodies (Alexa Fluor 488-labeled anti-rabbit, dilution 1:1000, and Alexa Fluor 647-labeled anti-mouse IgG1, dilution 1:1000; Thermo Fisher Scientific) with Alexa Fluor 594-conjugated α-bungarotoxin (dilution 1:1000, B13423; Thermo Fisher Scientific) for 48 h at 4°C. After final washing, tissues were flattened and mounted in Vectashield (Vector Laboratories, Inc., CA, USA).

**Table 1:** List of primary antibodies used for immunostaining.

Antibody	Dilution	Catalog. no.	RRID	Manufacturer	Reference
Anti-TH	1:200	AB152	AB_390204	Millipore	[25], [26]
Anti-neurofilament	1:1000	2H3	AB_531793	DSHB	[24], [27]
Anti-SV2	1:1000	SV2	AB_2315387	DSHB	[24], [28]

DSHB; Developmental Studies Hybridoma Bank

Confocal images were obtained using a Leica SP8 or STELLARIS WLL confocal microscope with 20 × HC PL FLUOTAR (NA = 0.4) or 40 × HC PL APO (NA = 1.30, oil CS2) objectives. Whole-mount immunohistochemically stained muscles were imaged as volumes using z-stacks with a step size of 0.25–1 μm, a digital zoom of 0.75–2 × , and sequential frame scanning for each channel to visualize thin sympathetic nerves and prevent channel bleed-through. Anti-TH staining patterns defined sympathetic nerves. Anti-neurofilament and anti-SV2 staining patterns defined motor nerve terminals. The nAChR clusters were determined by α-bungarotoxin staining patterns. The overlapping region between motor nerve terminals and nAChR defines NMJs.

#### Trace of TH-positive axons

We traced TH-positive axons in whole-mount-stained images to clarify the proximity of sympathetic branches around arterioles to NMJs. After deconvoluting the acquired images (Huygens, Scientific Volume Imaging, Holland or Lightning, Leica Microsystems, Germany), TH-positive axons were traced using the filament tracer tool of Imaris software (version 9.7.2, Bitplane, Belfast, UK). TH-positive axons were reconstructed using the AutoPath algorithm. In most cases, a single line from the starting point (artery) to the ending point (NMJ) could be used to detect the axon. If the axon could not be accurately followed, it was divided into consecutive sections and traced using the AutoPath algorithm.

### Physiological analysis

#### General preparation

Hindlimb muscle contractility and lumbar sympathetic nerve activity were recorded as described in our previous study [21]. In brief, animals were anesthetized with a subcutaneous injection of urethane (1.1 g/kg). During surgery and data collection, an additional dose (0.04–0.14 g/kg) was injected subcutaneously or intravenously whenever necessary. Sufficient anesthesia depth was determined based on loss of corneal and withdrawal reflexes and stable blood pressure and heart rate. Additionally, local anesthetics (procaine hydrochloride or lidocaine hydrochloride) were used at the incision site. The trachea was cannulated and connected to a ventilator (SN-480–7, Shinano, Tokyo, Japan) for artificial respiration with room air. The end-tidal CO_2_ level, measured by a gas monitor (Capnostream 35, Medtronic, Dublin, Ireland), was approximately 3.0 %. To monitor arterial blood pressure and inject phenylephrine and other drugs, catheters were inserted into the right femoral artery and vein. Body temperature, measured using a thermistor in the rectum, was maintained at 37.0°C–37.5°C using a thermostatically regulated heating sheet and lamp (ATC-101B-RS-S1, Unique Medical Co., Ltd, Tokyo, Japan). At the end of the experiment, animals were euthanized with a high dose of secobarbital sodium (50 mg per animal, i.v.).

To minimize the influence of blood catecholamines secreted from the adrenal glands and sympathetic nerves innervating the abdominal viscera, the major splanchnic nerves and adrenal plexus were crushed bilaterally [12]. Furthermore, in three rats, the left LST was cut at the L1–L2 levels to eliminate the activity of muscle sympathetic nerves innervating the gastrocnemius muscle. During surgery and experiment, 1 ml/kg of an isotonic electrolyte solution (Lactec D Injection, Otsuka Pharmaceutical Co., Ltd., Tokyo, Japan) was administered intravenously every hour to maintain body fluid levels and maintain a constant tetanic force in the hindlimb muscle.

#### Nerve stimulation to induce tetanic contractions of hindlimb muscles

The left tibial nerve was dissected from the surrounding tissue approximately 2.5 cm proximal to the knee joint. For electrical stimulation, the intact tibial nerve was placed on bipolar silver wire electrodes and embedded in silicon rubber (SIL604S-A and B, Wacker Asahikasei Silicone Co., Ltd., Tokyo, Japan). The left hindlimb was fixed, and electrical stimulation was applied to the tibial nerve to induce tetanic contractions while measuring the contraction force of the gastrocnemius and soleus muscle using a load cell (Ultra Small-Capacity Load Cell, LVS-20GA, Kyowa Electronics Instruments, Tokyo, Japan, 200 mN capacity) connected to the Achilles’ tendon with a thread. The obtained signal was amplified (AP-610J with MEG-6100, Nihon Kohden Co., Tokyo, Japan). We employed rectangular electrical pulses of 0.2 ms, with a stimulation current set to twice the motor threshold, which is subthreshold for group III and IV fibers [12]. Ten pulses of burst stimulation at 100 Hz, repeated 10 times every 1 s, were administered to induce a tetanic contraction. These 10 contractions were repeated three times at 1-min intervals, for a total of 30 contractions. Such intermittent stimulation was used to prevent fatigue in fast-fatiguing glycolytic muscle fibers [29], [30]. During surgery, care was taken to avoid stimulating the tibial nerve and stretching the surrounding muscles.

#### Recording activity in the sympathetic postganglionic nerve innervating the hindlimb

In four animals, mass nerve activity was recorded from a gray rami communicans of the lumbar segment, a specific pathway for sympathetic postganglionic fibers innervating the hindlimb. After excising the abdominal skin and muscle, nerve bundles from the caudal portion of the L2–L4 paraspinal sympathetic ganglion on the left side were slashed as close as possible to the spinal nerve and covered with liquid paraffin. Efferent discharge activity was recorded from the slashed central end of the nerve, placed on a bipolar platinum–iridium wire electrode, and amplified using a preamplifier (UA-200, Unique Medical Co., Ltd, Tokyo, Japan). To prevent possible artifact contamination, discharge activity was continuously monitored with an oscilloscope and speaker. The 200–1000-Hz band was extracted using Spike2 software (Cambridge Electronic Design, Cambridge, UK) and later rectified. Tibial nerve stimulation at twice the motor threshold produced little to no effect on sympathetic activity under muscle paralysis, as previously reported [21], [31]. Therefore, to observe sympathetic reflexes resulting from muscle contractions, animals were not paralyzed, even during nerve recordings.

#### Baroreceptor stimulation

Phenylephrine, a hypertensive drug, was administered intravenously to activate baroreceptors. Phenylephrine hydrochloride solution (Kowa Co., Ltd., Tokyo, Japan) was diluted with saline to a concentration of 160 μg/ml, and 1.4 ml/kg was administered. To achieve near-maximal arterial baroreceptor activation, mean arterial blood pressure (MAP) was maintained at approximately 150 mmHg by an initial bolus injection of phenylephrine solution (10 % of the total volume in 10 s); the remaining solution was administered continuously for 3 min. The same saline volume was administered in the same manner as phenylephrine. Drug injection lasted 190 s, starting 1 min before the onset of tibial nerve stimulation and finishing after the end of the stimulation. Before drug injection, sets of 30 tetanic contractions were repeated at 10-min intervals. When the amplitude of successive sets was stabilized, saline or phenylephrine was injected 2 min after the end of the control contraction set.

#### Removal of baroreceptor activity

The aortic nerve, including baroreceptor nerves from the aorta, was bilaterally transected to sever the neural pathway of the baroreceptor reflex. In eight of the nine rats, aortic nerves were bilaterally cut during the experiment. After denervation, the animals were allowed to recover for at least 20 min before further procedures.

#### Statistical analysis

All analog signals obtained were digitized (Micro 1401, Cambridge Electronic Design) and displayed on a computer monitor for online and offline analysis using Spike2 software. To quantify contractile force, waveforms from 30 tetanic contractions were averaged using the onset of tibial nerve train pulse stimuli as the trigger, and the peak amplitude of the averaged waveform was measured as the tetanic force. For the sympathetic reflex, rectified signals were averaged 30 times with the same trigger, and the area under the evoked excitatory response was measured as the reflex size. Mean arterial pressure was averaged over 130 s, from the onset to the offset of the train stimuli.

A paired *t*-test was used to compare values before and after injection. Changes induced by drug injections were expressed as percentage differences relative to control values before injection. Comparisons among three conditions were analyzed using one-way factorial analysis of variance (ANOVA), followed by the Fisher's Least Significant Difference (LSD) test for multiple comparisons (Prism 9, GraphPad Software, CA, USA). Simple linear regression was used to examine the relationship between tetanic force or sympathetic reflex and MAP responses. Data are presented as mean ± SD unless otherwise stated. Statistical significance was set at p < 0.05.

## Results

### Physical connection of TH-immunoreactive sympathetic axons from the artery to the NMJ in gastrocnemius muscle

**Fig. 1:**
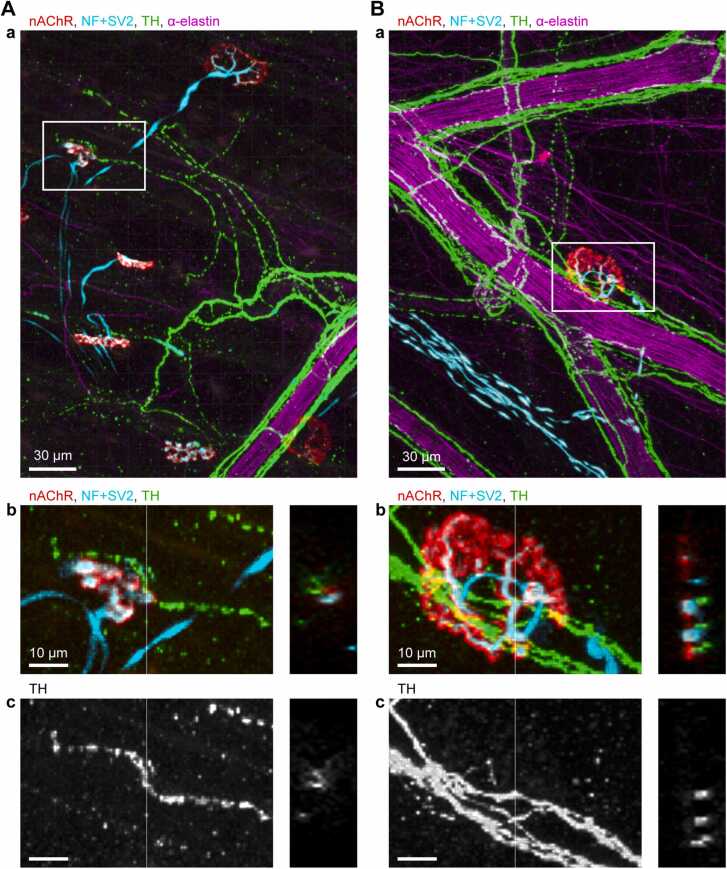
**Sympathetic nerves innervating an artery appear to connect to the neuromuscular junction (NMJ) in rat gastrocnemius muscle.** A, B: Two representative confocal images showing sympathetic nerves (green, stained with anti-TH antibodies), arteries (magenta, visualized with α-elastin), and NMJs with motor nerves (blue, stained with anti-neurofilament and anti-SV2 antibodies) innervating muscle endplates (red, labeled with fluorescent α-bungarotoxin binding to acetylcholine receptors). a: Three-dimensional (3D) reconstructions spanning depths of 39–86 μm were generated from z-stack images acquired at 1-μm intervals and collapsed into maximum-intensity projections. Channel intensity and contrast were linearly adjusted to prepare RGB images. b, c: Enlarged view of the white boxed region in (a). The gray scale images in (c) present the TH signal. The right panel shows an orthogonal section along the vertical line indicated in the left panel.

The connection between sympathetic nerves and the NMJ was examined using 3D images (z-stack thickness: 40–161 μm) obtained from whole-mount specimens of rat gastrocnemius muscle. Fig. 1Aa shows a representative maximum-intensity projection image of immunohistochemical staining, visualizing endplates with nAChRs, motor nerves, sympathetic nerves, and arteries. Colocalization of motor nerve terminals with nAChRs indicated the NMJ site. Additionally, a striated TH signal around an α-elastin structure confirmed that sympathetic nerves ran along the artery. From this artery, a TH-positive axon branch was observed extending to an NMJ. 3D analysis was performed to examine the structure from all angles. Tracing software identified the axon branch connecting the artery to the NMJ, measuring 274 μm in length (highlighted in yellow in the 3D reconstruction, Movie 1). The TH signal was strong around the artery, whereas the TH-positive axon extending toward the NMJ typically appeared thinner and weaker in intensity. Fig. 1Ab and 1Ac present an enlarged view with an orthogonal section, showing that the TH-positive axon lies in close proximity to both presynaptic motor nerve terminals and postsynaptic nAChRs of the NMJ.

Fig. 1Ba shows another example in which an NMJ was located close to sympathetic varicosities around an artery. The enlarged NMJ with an orthogonal section view (Fig. 1Bb, Bc) and the corresponding 3D reconstruction (Movie 2) demonstrated that TH-positive axons appeared to align with motor nerve terminals and nAChRs.

We identified a total of 13 instances of sympathetic axons physically connecting arteries to NMJs (n = 4 rats, 2–4 axons per rat), as summarized in Table 2. The traced connecting axon lengths ranged from 0 μm (Fig. 1B) to 418 μm.

**Table 2:** Measurements of axon length from artery to NMJ and size of nAChR cluster.

Trace #	Axon length (artery to NMJ)	Longitudinal axis length of nAChR cluster
1	84.6	25.7
2	418	20
3	125	21.5
4	122	21.8
5	257	22
6	162	24.6
7	196	34.6
8	306	26.5
9	159	22.2
10	0	43.3
11	274	21.3
12	0	39.4
13	178	37.1

Values are presented in μm. A value of 0 for axon length indicates that the NMJ was located adjacent to a sympathetic axon around an artery.

### Arterial baroreceptor activation reduced tetanic force in gastrocnemius and soleus muscles

#### Intact aortic nerve

The effects of arterial baroreceptor activation on tetanic contractions of the gastrocnemius and soleus muscles induced by tibial nerve stimulation were examined in four rats with intact aortic nerves. In these rats, basal MAP levels ranged from 45 to 95 mmHg. In this experiment, the splanchnic nerves were cut to minimize humoral influences from sympathetic terminals in the abdominal viscera, thereby maintaining relatively low baseline arterial pressure and minimal basal aortic activity [32]. Arterial baroreceptors were activated to near-maximal levels by a rapid increase in MAP induced by intravenous phenylephrine injection.

**Fig. 2:**
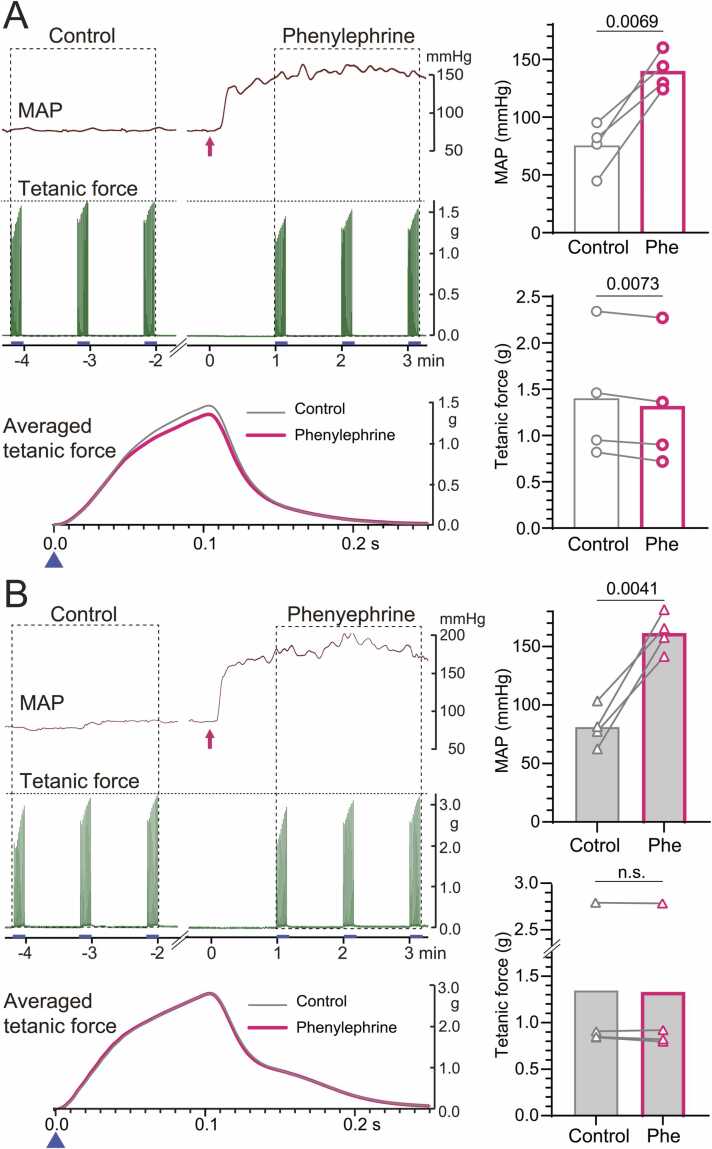
**Effects of phenylephrine injection on MAP and tetanic force of the gastrocnemius and soleus muscles in rats with (A) and without (B) aortic nerve innervation.** Left: Representative recordings of mean arterial pressure (MAP) and tetanic force monitored simultaneously before (control) and after intravenous phenylephrine injection. The arrow indicates the start of injection. The lower trace shows the averaged tetanic force (30 trials) before (control) and during phenylephrine injection. Zero (▲) on the X-axis represents the onset of tetanic tibial nerve stimulation (0.2 ms, 2 × motor threshold, 10 pulses at 100 Hz). Right: Summary graphs showing MAP and tetanic force values before and after injection in four rats. Bars indicate mean values; each symbol with its connecting line represents data from an individual rat.

Fig. 2A shows a representative example of tetanic contractions and MAP in the pre-injection control condition and those recorded following phenylephrine administration. After injection, MAP increased rapidly, reaching peak levels in approximately 60 s; this increased level continued during phenylephrine administration (Fig. 2A, upper trace). Conversely, the maximum force observed during phenylephrine injection was lower than that in the control (middle trace in Fig. 2A). Then, force waveforms were averaged across all 30 repetitions in each condition and superimposed (lower trace in Fig. 2A). During phenylephrine administration, the peak time of force generation did not change, but the peak amplitude was slightly lower than that of the control. Similar results were obtained in all four rats tested. Following phenylephrine injection, MAP increased significantly from 73.1 ± 16.8 mmHg to 139.8 ± 12.5 mmHg (p = 0.0069), whereas tetanic force decreased significantly from 1.35 ± 0.60 g to 1.29 ± 0.59 g (p = 0.0073) (right graphs in Fig. 2A). No changes in MAP or tetanic force were observed when saline was administered instead of phenylephrine, tested in four animals.

**Fig. 3:**
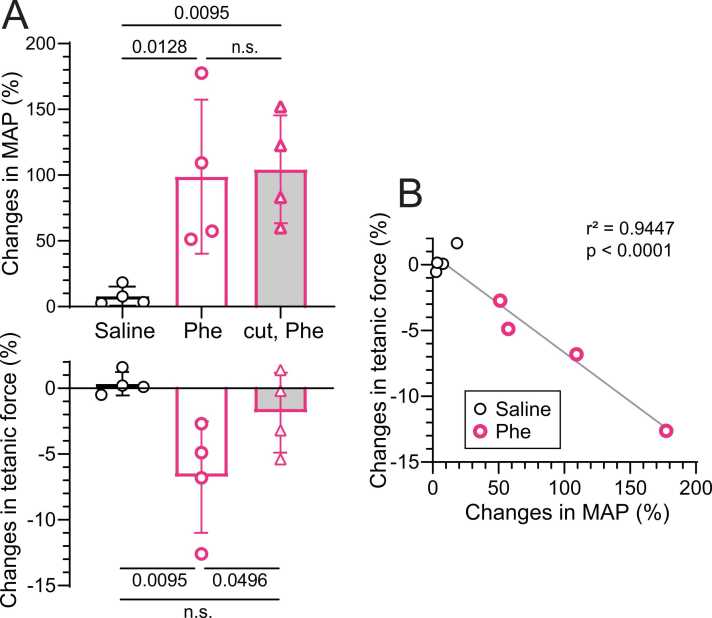
**Percent changes in MAP and tetanic force of the gastrocnemius and soleus muscles induced by phenylephrine.** A: Percent changes in tetanic force and MAP induced by saline or phenylephrine (Phe) in rats with intact (open columns) or transected (gray columns) aortic nerves. A value of 100 % represents the control (before injection). Bars and vertical lines indicate mean ± SD (n = 4 rats). Each symbol represents data from an individual rat. B: Relationship between the magnitude of changes in MAP and tetanic force during phenylephrine or saline injection in rats with intact aortic nerves.

Fig. 3A summarizes the changes in tetanic force and MAP, expressed as percent changes relative to control values before injection. Tetanic force decreased significantly by 6.8 % ± 4.2 % (range, 2.7 %–12.6 %; p = 0.0095) following phenylephrine injection compared with saline infusion. Conversely, MAP increased significantly by 99 % ± 59 % (range, 51 %–178 %; p = 0.0128) during phenylephrine injection compared with saline injection (Fig. 3A). When the magnitude of the tetanic force response was plotted against that of the MAP response to intravenous phenylephrine or saline injection, a strong correlation was observed between the magnitude of force reduction and the magnitude of MAP increase (r^2^ = 0.9447, p < 0.0001; Fig. 3B).

In the other three rats in which the LST was transected, MAP increased from 57 ± 15 mmHg to 123 ± 28 mmHg with phenylephrine (p = 0.0140), whereas tetanic force remained unchanged (p = 0.728) (Additional Fig. 1). These results indicate that lumbar sympathetic nerve activity is essential for the tetanic force response.

#### Denervated aortic nerve

The effect of baroreceptor activation on tetanic force was examined in four rats without aortic nerve innervation. In these denervated rats, phenylephrine significantly (p = 0.0041) increased MAP by 104 % ± 41 % during administration, similar to that observed for intact aortic nerve. However, the decreased tetanic force observed in rats with intact aortic nerves was abolished (Fig. 2B). When aortic nerves were cut bilaterally, tetanic force amplitude was not affected by intravenous phenylephrine injection. The changes in tetanic force after phenylephrine administration differed significantly between intact and cut aortic nerves (p = 0.0496; Fig. 3A, lower graph), although changes in MAP did not differ irrespective of aortic nerve innervation (Fig. 3A, upper graph).

### Arterial baroreceptor activation inhibited the excitatory musculo–lumbar sympathetic reflex discharge

#### Intact aortic nerve

At the beginning of each experiment, we confirmed the induction of sympathetic reflex discharges in postganglionic fibers of the gray rami communicantes in the lumbar segment by stimulating the tibial nerve to induce muscle contraction in anesthetized rats, as described by Hotta et al. [21].

**Fig. 4:**
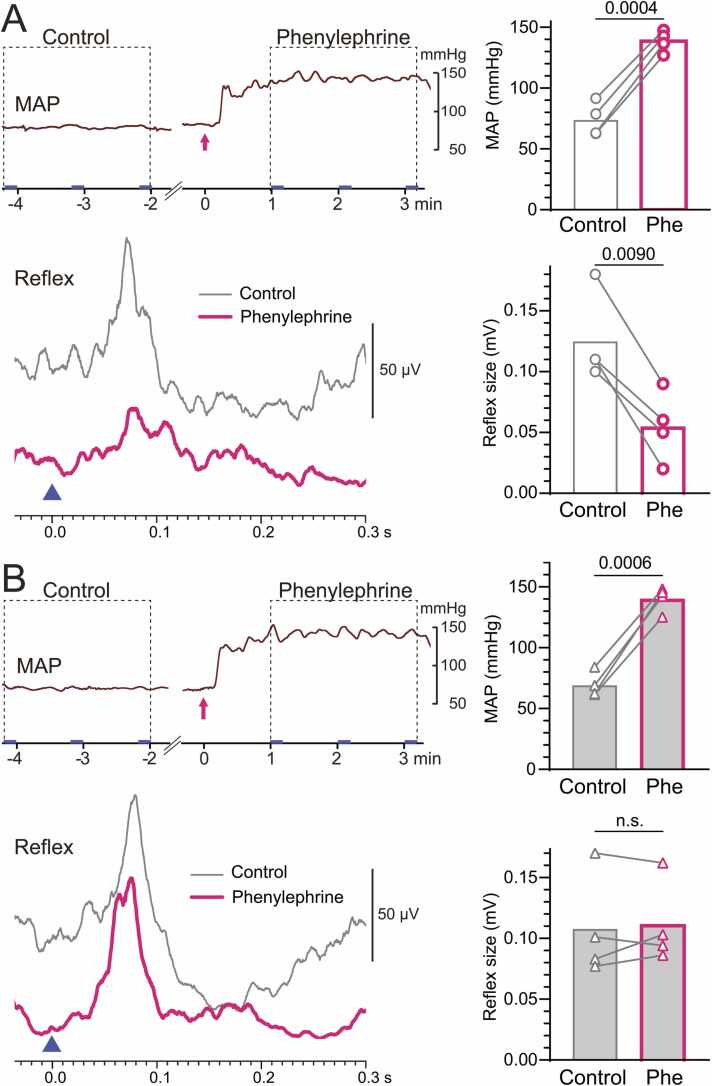
**Effects of phenylephrine injection on excitatory musculo–lumbar sympathetic reflex discharges in rats with (A) and without (B) aortic nerve innervation.** Left: Representative recordings of MAP and musculo–lumbar sympathetic reflex (averaged 30 times; mean of 4 trials in 4 rats) before (control) and during intravenous phenylephrine injection in rats with intact aortic nerves. Zero (▲) on the X-axis indicates the onset of tetanic tibial nerve stimulation (0.2 ms, 2 × motor threshold, 10 pulses at 100 Hz). Right: Summary graphs showing MAP and musculo–lumbar sympathetic reflex before and after injection in four rats. Bars indicate mean values; each symbol with its connecting line represents data from an individual rat.

Fig. 4A shows a representative example of the contraction-induced sympathetic reflex and MAP recorded before (control) and after phenylephrine injection. Following phenylephrine injection, MAP increased, whereas the sympathetic reflex discharge response was markedly depressed (p = 0.009; Fig. 4A). In contrast, reflex amplitude and MAP showed minimal changes with saline administration.

**Fig. 5:**
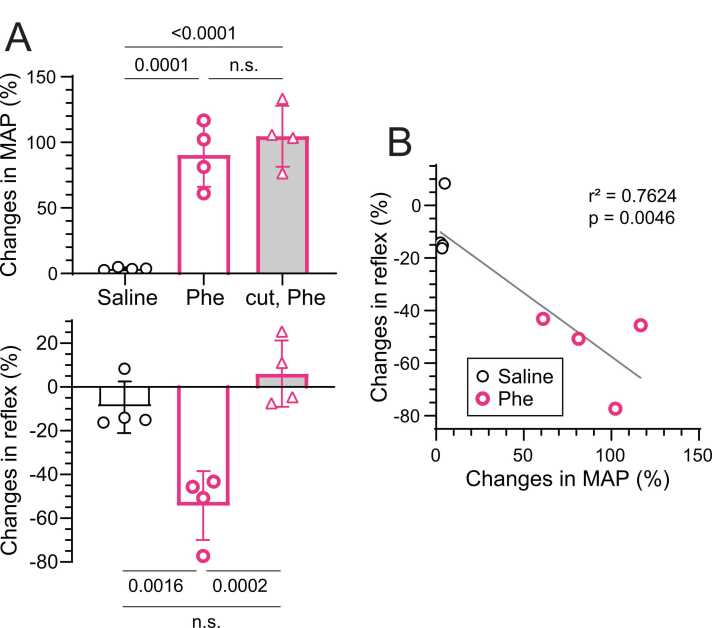
**Percent changes in MAP and excitatory musculo–lumbar sympathetic reflex discharges induced by phenylephrine.** A: Percent changes in musculo–lumbar sympathetic reflex and MAP induced by saline or phenylephrine (Phe) in rats with intact (open columns) or transected (gray columns) aortic nerves. A value of 100 % represents the amplitude before injection (control). Bars and vertical lines indicate mean ± SD (n = 4 rats). Each symbol represents data from an individual rat. B: Relationship between MAP and reflex magnitude during drug injection in rats with intact aortic nerves.

Fig. 5A summarizes the changes in reflex response after injection, expressed as a percentage of the control response recorded before injection. The reflex response decreased by 54 % ± 16 % (range, 43 %–77 %) following phenylephrine injection, a significantly greater reduction than that observed after saline injection (p = 0.0016). MAP increased by 90 % ± 24 % (range, 61 %–117 %) during phenylephrine injection compared with saline injection (p = 0.0001). Plotting the relationship between arterial pressure levels and the musculo–lumbar sympathetic reflex measured in four animals during intravenous phenylephrine or saline injection indicated that reflex magnitude decreased with increasing MAP with a significant negative correlation (r^2^ = 0.7624, p = 0.0046; Fig. 5B).

#### Denervated aortic nerve

The effect of baroreceptor activation on the musculo–lumbar sympathetic reflex was examined in four rats after bilaterally denervating the aortic nerve. In denervated rats, phenylephrine injection increased MAP by 97 % ± 14 % (p < 0.0001), similar to that obtained with intact aortic nerves. However, the reflex response depression observed in rats with intact aortic nerves was abolished, with no significant change in the magnitude of reflex response, as shown in a representative recording in Fig. 4B. The changes in reflex amplitude following phenylephrine differed significantly between intact and cut aortic nerves (p = 0.0002). However, MAP changes did not differ between aortic nerve innervation conditions (Fig. 5A).

### Effect of baroreceptor activation on tonic activity of the lumbar sympathetic nerve in rats with and without aortic nerve innervation

**Fig. 6:**
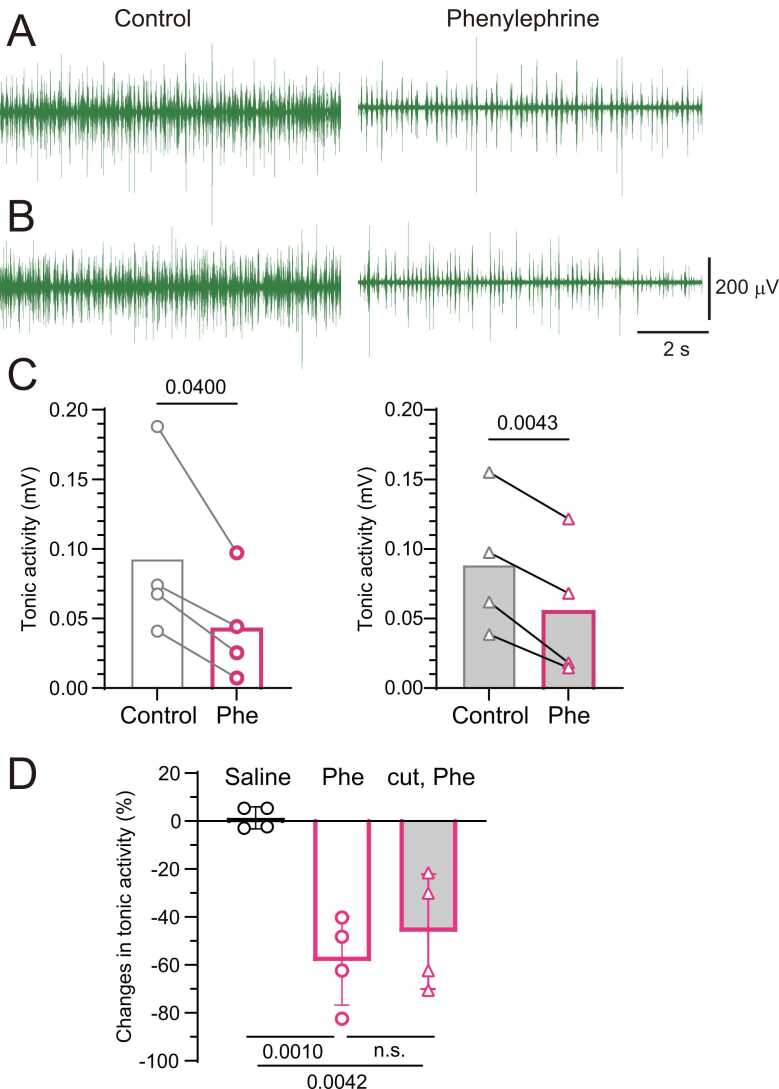
**Effects of phenylephrine injection on lumbar sympathetic nerve tonic activity.** A, B: Representative original recordings of lumbar sympathetic nerve activity before (control) and after intravenous phenylephrine injection in the same rat with (A) and without (B) aortic nerve innervation. C: Summary graphs of sympathetic activity before and after injection in four rats with (left) and without (right) aortic nerve innervation. D: Percent changes in tonic lumbar sympathetic nerve activity induced by saline or phenylephrine (Phe) in rats with intact (open column) or transected (gray column) aortic nerves. A value of 100 % represents the mean activity for 1 min before drug injection. Bars and vertical lines indicate mean ± SD (n = 4 rats). Each symbol represents data from an individual rat.

The basal activity of the lumbar sympathetic nerve without tibial nerve stimulation was compared before and after intravenous phenylephrine injection. The mean activity during the 1 min preceding phenylephrine injection was used as the control. The mean activity over 10 s, taken 50–60 s after the onset of phenylephrine injection when MAP reached near maximum and the tibial nerve was not stimulated, was expressed as a percentage of the control. As shown in the original recordings in Figs. 6A and 6B, tonic discharge decreased following phenylephrine injection, both with (A) and without (B) aortic nerve innervation. The decrease in tonic discharge was consistently observed across all animals in both aortic nerve conditions (Fig. 6C). In four trials in four rats with aortic nerve innervation, the average discharge of lumbar sympathetic nerves decreased by 58 % ± 18 % of the control after phenylephrine injection (p = 0.0010 vs. saline, Fig. 6D). In rats without aortic nerve innervation, the decrease in tonic activity was 46 % ± 24 % (p = 0.0042 vs. saline), which was not significantly different from that in rats with intact aortic nerves.

## Discussion

In this study, we fluorescently labeled arteries, sympathetic and motor nerves, and nAChRs and showed that some sympathetic axons innervating arteries extend to the NMJ via whole-mount preparation of gastrocnemius muscles. Using fluorescence and electron microscopy in cat hindlimbs deprived of somatic innervation, Barker and Saito [33] reported that some adrenergic axons innervating skeletal muscle fibers are axon branches that also innervate blood vessels. However, they did not observe NMJs. More recently, Straka et al. [14] performed a preliminary analysis of cross-sections of different hindlimb muscles showing that TH-positive structures mostly contact NMJs (α-bungarotoxin) and blood vessels (isolectin-B4). However, they did not visualize arteries to demonstrate the sympathetic nerve connection between arteries and NMJs. Our histological analysis suggested the possible existence of adrenergic nerves innervating both an NMJ and an artery.

The significant, novel finding of this study was that aortic baroreceptor activity affects motor nerve-induced muscle contractility, a discovery with important practical implications. Specifically, activation of aortic nerves via intravenous phenylephrine injection induced a modest reduction in tetanic force (6.8 %) in a blood pressure–dependent manner. The abolition of this reduction after bilateral aortic nerve transection further supports the role of baroreflexes mediated by aortic nerve activation. A similar disappearance of the tetanic force reduction was observed after transection of the LST at the L1–L2 level, indicating that the efferent pathway involves the lumbar sympathetic nerve. We previously demonstrated that tetanic force decreases when transecting the LST, and a similar decrease in tetanic force occurred when the afferent pathway (lumbar dorsal roots), but not the efferent pathway (LST), was interrupted, suggesting that the excitatory musculo–lumbar sympathetic reflex induced by muscle contractions was involved in modulating their contractility [21].

In the present study, postganglionic sympathetic nerve activity to the hindlimb was recorded to examine whether the reflex potential elicited in response to contractions was inhibited by arterial baroreceptor activation. We showed that activation of arterial baroreceptors by a 90 % increase in arterial pressure, induced by intravenous phenylephrine injection, resulted in 54 % suppression of the excitatory musculo–lumbar sympathetic reflex in anesthetized rats. This result corroborated with a previous study reporting a 50 %–60 % suppression of somato–cardiac sympathetic A and C reflexes with a 100 % increase in arterial pressure due to phenylephrine administration [4]. Similar arterial pressure–dependent reductions were reported in exercise pressor reflexes in both conscious humans [34] and anesthetized dogs [35]. We further demonstrated that with bilaterally transected aortic nerves, the inhibition of sympathetic reflex discharge disappeared after intravenous phenylephrine injection, although the systemic arterial blood pressure remained elevated. Thus, the suppression of excitatory sympathetic reflex discharge after intravenous phenylephrine injection is a reflex involving the aortic nerve as an afferent pathway.

The degree of inhibition of sympathetic tonic activity was comparable to that of the musculo–lumbar sympathetic reflex when the aortic nerves were intact. However, after bilateral aortic nerve transection, the inhibitory effect on lumbar sympathetic nerve tonic activity persisted. Previous studies considered it improbable that phenylephrine had a direct inhibitory effect on sympathetic neurons [4], [5]. Thus, the remaining baroreceptor signaling pathways, such as the carotid sinus nerves in this case, would be responsible for suppressing the tonic activity, as reported for renal and general vasoconstrictors [5], [36]. Our results demonstrated that the inhibition of excitatory musculo–lumbar sympathetic reflex discharges induced by intravenous phenylephrine injection was independent of sympathetic tonic activity.

The hypertension-induced changes in tetanic force disappeared after severing aortic nerves, similar to changes in the musculo–lumbar sympathetic reflex but different from those in sympathetic tonic activity, which remained inhibited even without aortic nerve innervation. These results indicate that the magnitude of the musculo–lumbar sympathetic reflex, rather than the level of background tonic activity, determines the end organ response. Muscle sympathetic tonic activity, which contributes significantly to maintaining basal vascular tone, appears to be less critical for regulating tetanic force. Modulation of the musculo–lumbar sympathetic reflex through the level of afferent input from aortic baroreceptors may account for baroreceptor-dependent adjustments in muscle contractility. Therefore, an interaction between the baroreceptor input from the aorta and the somatosensory input from contracting muscles is essential. The musculo–lumbar sympathetic reflex, observed in an intact central nervous system, is supraspinal reflex [21] that is possibly mediated by neurons in the rostral ventrolateral medulla (RVLM) [37], [38]. The musculo–sympathetic reflex, mediated by group III and group IV afferents from skeletal muscle, is responsible for circulatory system adaptation to exercise; a mechanism originating from intramuscular chemosensors or ergoreceptors and mediated by RVLM neurons [39]. Group III and group IV somatic afferent inputs from contracting muscles reportedly interacted with the baroreceptor input at the brainstem nucleus, such as the nucleus tractus solitarius [40], [41], [42], [43].

### Limitations

Our histological study does not rule out the possibility of sympathetic nerves innervating NMJs independently of arterial innervation. However, our functional results indicated that tetanic force decreased by 6.8 % when the reflex response of the mass sympathetic discharge was partially (by 54 %) attenuated due to baroreceptor activation; this result is consistent with our previously reported results that tetanic force decreased by ∼10 % after LST transection [21], [22]. Therefore, most sympathetic nerves innervating NMJs appear to produce the same type of responses as vasomotor fibers, consistent with our histological observation that TH-positive fibers innervate both arteries and NMJs. However, future studies are needed to examine whether removing sympathetic synapses at the NMJ abolishes baroreceptor modulation of tetanic force.

The tibial nerve was stimulated to evoke tetanic contraction. This stimulation activates not only motor nerves but also afferent nerves. The stimulus intensity we used (i.e., twice the motor threshold) was subthreshold for the thin myelinated and unmyelinated afferent fibers that generate sympathetic reflexes. However, we cannot exclude the potential confounding effects of stimulating thick myelinated afferent nerve fibers.

In this study, systemic pharmacological intervention was used to stimulate arterial baroreceptors by vasoconstriction. Therefore, there may be confounding effects of the drug on tissues other than vascular smooth muscles. However, we observed a clear difference in tetanic force and musculo–lumbar sympathetic reflex responses between drug injection before and after aortic nerve transection, suggesting that the baroreceptor reflex is the main effect. Tetanic force decreases in parallel with reduced blood flow in slowly fatiguing aerobic muscle fibers, but not in fast-fatiguing fibers [29]. Notably, our method primarily evaluated the force of fast-fatiguing muscle fibers.

Based on our results, we cannot determine whether the noradrenaline released from sympathetic terminals facilitates motor nerve function at NMJs by acting on presynaptic motor terminals or postsynaptic sarcolemma. Further, the short burst stimulation of 10 impulses at 100 Hz used in this study mimics motor neuron activity in conscious rats [44]. Pharmacological studies suggested complexity of adrenergic regulation of NMJs and that various types of adrenergic receptors can facilitate ACh release [45]. For example, acute pharmacological activation of β2-adrenoceptors enhances neuromuscular transmission during intense activity of 20–100 Hz by increasing the recruitment of synaptic vesicles into the evoked exocytosis [46]. Such adrenergic regulation may be an essential component for enhancing muscle performance, particularly during intense/prolonged activity, or in neuromuscular diseases, when the safety factor of neurotransmission decreases ( [47], for a review of the Safety factor).

## Conclusion

**Fig. 7:**
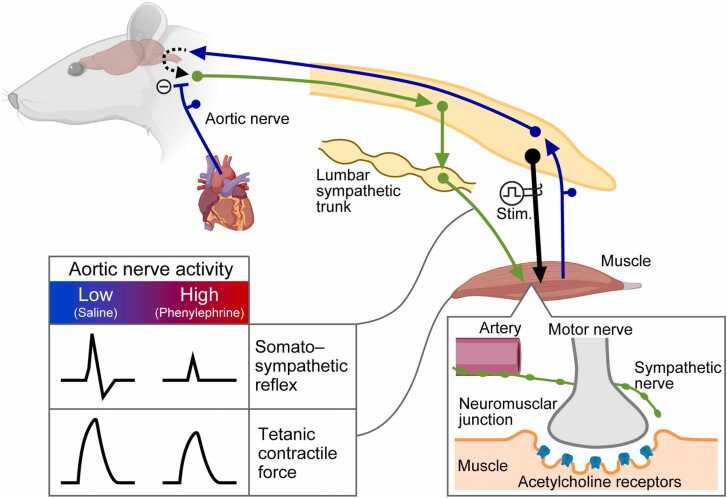
**Schematic diagram summarizing the present findings.** The tetanic contraction of hindlimb muscles induced by motor nerve stimulation (Stim.) produces an excitatory somato–lumbar sympathetic reflex. The excitatory reflex is inhibited by baroreceptor input from the aortic nerve, thereby modifying the degree of sympathetic support for tetanic force generation (bottom left). The histological finding that some sympathetic nerve axons connect from the artery to both presynaptic motor nerve terminals and postsynaptic acetylcholine receptor clusters in the neuromuscular junction (bottom right) supports this explanation. Created using BioRender.com.

Some muscle sympathetic nerves innervating arteries also project to NMJs, where nAChR clusters and motor nerve endings are colocalized. During hindlimb muscle contraction induced by motor nerve tetanic stimulation, somatic afferent input from the muscles triggers an excitatory somato–sympathetic reflex that enhances tetanic contractile force. Baroreceptor input from the aortic nerve inhibits this excitatory somatic afferent input, likely at the brainstem level, thereby modulating the extent of sympathetic support for motor nerve function (Fig. 7). Sympathetic nerves distributed to arteries and NMJs may underlie this modulation.

## Author contributions

H.H. conceived and designed the research. N.W., M.M., and H.H. performed physiological experiments. K.T., N.T., N.W., and H.N. performed histology and imaging. N.W. and H.H. performed analyses. H.H. wrote the paper with input from all authors. H.H. supervised the work.

## CRediT authorship contribution statement

**Kotaro Takeno:** Writing – review & editing, Data curation. **Nobuhiro Watanabe:** Writing – review & editing, Visualization, Methodology, Formal analysis, Data curation. **Harumi Hotta:** Writing – original draft, Funding acquisition, Formal analysis, Data curation, Conceptualization. **Hiroshi Nishimune:** Writing – review & editing, Visualization, Methodology, Data curation. **Masamichi Moriya:** Writing – review & editing, Data curation. **Naoko H. Tomioka:** Writing – review & editing, Formal analysis, Data curation.

## Funding sources

This work was supported by the JSPS-KAKENHI grant number 23K20363 (H.H.).

## Declaration of Competing Interest

The authors declare the following financial interests/personal relationships which may be considered as potential competing interests: Harumi Hotta reports financial support was provided by Japan Society for the Promotion of Science. Harumi Hotta serves as a chief editor of the Journal of Physiological Sciences. Given her role, she had no involvement in the peer review of this article and had no access to information regarding its peer review. Full responsibility for the editorial process for this article was delegated to another journal editor. The other authors declare that they have no known competing financial interests or personal relationships that could have appeared to influence the work reported in this paper.

## Data Availability

The data supporting the findings of this study are available from the corresponding author upon reasonable request.

## Appendix A Supplementary material

Supplementary material

**Movie 1. Tracing of a TH-positive axon between an artery and a neuromuscular junction.** A TH-positive axon traced between an artery and a neuromuscular junction is highlighted in yellow. The movie was constructed from the three-dimensional image shown in Fig. 1A

**Movie 2. Three-dimensional reconstruction of TH-positive axons around an artery and a neuromuscular junction.** This movie was constructed from the three-dimensional image shown in Fig. 1B
