# Supplementary material for: Activation of aortic baroreceptors depresses the somato–lumbar sympathetic reflex, reducing hindlimb muscle contractile force
Source: J Physiol Sci. 2025 Nov 20;76(1):100051. doi: 10.1016/j.jphyss.2025.100051 (PMC12701687; doi:10.1016/j.jphyss.2025.100051)
Supplement: Supplementary file 1 — Supplementary material [file mmc1.docx]

**Supplementary material**


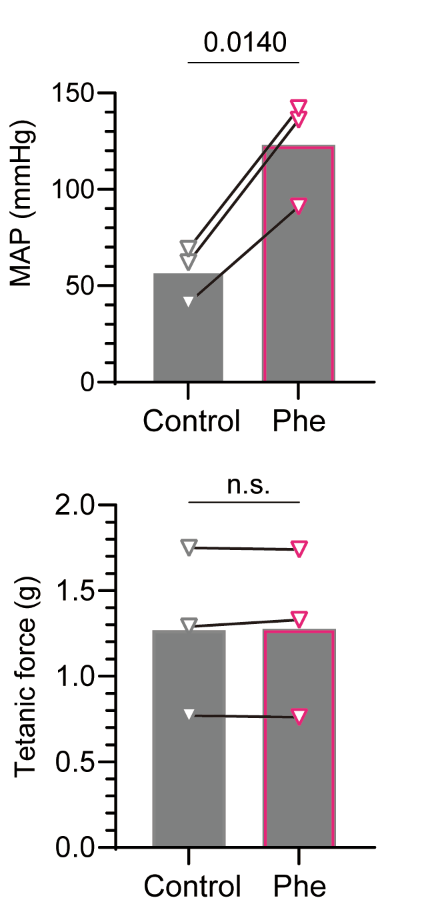


**Additional Figure 1. Effects of phenylephrine injection on mean arterial pressure (MAP) and tetanic force of the gastrocnemius and soleus muscles after lumbar sympathetic trunk transection.**

MAP and tetanic force were monitored simultaneously before (control) and after intravenous phenylephrine injection in rats with lumbar sympathetic trunk transection (n = 3). MAP and tetanic force values are summarized in the graphs. Bars indicate mean values, and each symbol with its connecting line represents data from an individual rat.
